# Diabetes management in patients undergoing total pancreatectomy: A single center cohort study

**DOI:** 10.3389/fendo.2023.1097139

**Published:** 2023-02-13

**Authors:** Tianyi Zhao, Yong Fu, Taiping Zhang, Junchao Guo, Quan Liao, Shuoning Song, Yanbei Duo, Yuting Gao, Tao Yuan, Weigang Zhao

**Affiliations:** ^1^ Department of Endocrinology, Key Laboratory of Endocrinology of Ministry of Health, Peking Union Medical College Hospital, Chinese Academy of Medical Science and Peking Union Medical College, Beijing, China; ^2^ Department of General Surgery, Peking Union Medical College Hospital, Chinese Academy of Medical Sciences and Peking Union Medical College, Beijing, China

**Keywords:** total pancreatectomy, pancreatogenic diabetes, diabetes management, insulin pump, continuous glucose monitoring

## Abstract

**Background:**

Total pancreatectomy (TP) has been increasingly performed in recent years. However, studies on diabetes management after TP during different postoperative periods are still limited.

**Objectives:**

This study aimed to evaluate the glycemic control and insulin therapy of patients undergoing TP during the perioperative and long-term follow-up period.

**Methods:**

Ninety-three patients undergoing TP for diffuse pancreatic tumors from a single center in China were included. Based on preoperative glycemic status, patients were divided into three groups: nondiabetic group (NDG, n = 41), short-duration diabetic group (SDG, preoperative diabetes duration ≤12 months, n = 22), and long-duration diabetic group (LDG, preoperative diabetes duration >12 months, n = 30). Perioperative and long-term follow-up data, including the survival rate, glycemic control, and insulin regimens, were evaluated. Comparative analysis with complete insulin-deficient type 1 diabetes mellitus (T1DM) was conducted.

**Results:**

During hospitalization after TP, glucose values within the target (4.4-10.0 mmol/L) accounted for 43.3% of the total data, and 45.2% of the patients experienced hypoglycemic events. Patients received continuous intravenous insulin infusion during parenteral nutrition at a daily insulin dose of 1.20 ± 0.47 units/kg/day. In the long-term follow-up period, glycosylated hemoglobin A1_c_ levels of 7.43 ± 0.76% in patients following TP, as well as time in range and coefficient of variation assessed by continuous glucose monitoring, were similar to those in patients with T1DM. However, patients after TP had lower daily insulin dose (0.49 ± 0.19 vs 0.65 ± 0.19 units/kg/day, *P* < 0.001) and basal insulin percentage (39.4 ± 16.5 vs 43.9 ± 9.9%, *P* = 0.035) than patients with T1DM, so did those using insulin pump therapy. Whether in the perioperative or long-term follow-up period, daily insulin dose was significantly higher in LDG patients than in NDG and SDG patients.

**Conclusions:**

Insulin dose in patients undergoing TP varied according to different postoperative periods. During long-term follow-up, glycemic control and variability following TP were comparable to complete insulin-deficient T1DM but with fewer insulin needs. Preoperative glycemic status should be evaluated as it could guide insulin therapy after TP.

## Introduction

More recently, postoperative outcomes following total pancreatectomy (TP) have improved with the advances in surgical techniques, glycemic monitoring, insulin delivery systems, insulin formulations, and pancreatic enzyme preparations (1–5). Recent series also demonstrated that TP was not inferior to pancreaticoduodenectomy regarding mortality, major morbidity, overall quality of life, and long-term survival (6–10). In addition, the major indications for TP have expanded to a range of diffuse pancreatic diseases over decades, encompassing pancreatic carcinoma with repeated positive frozen section margin, intraductal papillary mucinous neoplasm (IPMN), multifocal pancreatic neuroendocrine tumor (PNET), multifocal pancreatic metastases and chronic pancreatitis (4, 9, 11, 12). Consequently, TP has been performed increasingly in recent years (1, 3, 4, 13).

Diabetes after TP is characterized by the complete deficiency of insulin, pancreatic glucagon, and pancreatic polypeptide. Lund et al. (14) described the hormone profiles of 10 TP patients, showing undetectable plasma levels of C-peptide and pancreatic polypeptide but detectable concentrations of gut-derived fasting glucagon. Robust postprandial responses in glucagon induced by the rapid intestinal transit time were also revealed, leading to poor postprandial glucose control in TP patients (14). Besides, TP for neoplasms usually involves resection of part of the upper gastrointestinal tract, such as the distal stomach and duodenum, resulting in gastrointestinal motility disorders, rapid food transit, and lack of gut hormones, which together with malabsorption and fatty diarrhea due to pancreatic exocrine insufficiency further affects stable nutrient absorption and glycemic stability. Adjuvant chemotherapy, glucocorticoids, long-acting somatostatin analogs, and tyrosine kinase inhibitors for the treatment of underlying disease also had their potential negative influence on glycemic control. Moreover, a retrospective cohort study identified early postoperative fasting blood glucose as one of the independent risk factors for postoperative complications in patients undergoing TP, and high postoperative glycosylated hemoglobin A_1c_ (HbA_1c_) was associated with poor recurrence-free survival and overall survival (15).

Diabetes after TP has a significant impact on patients’ metabolic status, and good glycemic control is crucial for improving short- and long-term outcomes. However, detailed descriptions of diabetes management following TP during different postoperative periods remain limited. In the present study, we established a large cohort of patients undergoing TP for diffuse pancreatic tumors to summarize the characteristics of diabetes secondary to TP during perioperative and long-term follow-up, including the continuous glucose monitoring (CGM) parameters and insulin pump therapy which were rarely reported before, and compared these with complete insulin-deficient type 1 diabetes mellitus (T1DM).

## Methods

### Study population

Ninety-three patients who underwent TP with at least a 3-month follow-up in Peking Union Medical College Hospital between January 2009 and April 2022 were enrolled in the cohort. Excluded were those who deceased or withdrew within three months after surgery. A total of 10, 28, and 34 cases of TP were performed from 2009 to 2012, 2013 to 2016, and 2017 to 2020, respectively, and 21 cases were conducted since 2021. All 93 patients were candidates for TP due to diffuse pancreatic tumors confirmed by preoperative computed tomography or magnetic resonance imaging. Eighty-seven patients underwent one-stage TP, and six underwent completion TP for tumor occurrence or postoperative bleeding, including four who underwent distal pancreatectomy after pancreaticoduodenectomy and two who underwent pancreaticoduodenectomy after distal pancreatectomy. This study was approved by the Ethics Committee of Peking Union Medical College Hospital (JS-2574).

### Perioperative management

Data on demography, pathology, postoperative complications, length of stay, and preoperative glycemic status were collected in the 93 patients underwent TP. All patients were treated with total parenteral nutrition (TPN) and continuous intravenous insulin infusion (CVII) within the first few days after surgery. The insulin infusion rate was started at 0.05-0.10 units/kg/h and was adjusted or discontinued according to the TPN rate and blood glucose levels to reach a target glucose level. Subcutaneous long-acting insulin in combination with CVII was prescribed for some patients with a longer duration of parenteral nutrition. Parenteral nutrition was terminated when the patients resumed enough oral intake. The insulin regimens at different periods (parenteral nutrition, enteral nutrition, and one month after surgery) and daily carbohydrate content of parenteral nutrition were documented. Peripheral blood glucose was measured every 2 to 6 hours, depending on the glucose status. We recorded all the in-hospital glucose data to evaluate the individual’s mean and standard deviation (SD) of blood glucose per day, daily coefficient of variation (CV), maximum blood glucose, minimum blood glucose, and 6 am blood glucose. The target range for blood glucose in the perioperative period was defined as 4.4 to 10.0 mmol/L (16). Hypoglycemia was a blood glucose value of less than 3.9mmol/L according to the guideline for inpatient glucose management (16). Body mass index (BMI) was calculated as weight in kilograms divided by the square of the height in meters. The definition of CV (%) was 100 times the mean blood glucose divided by the SD.

### Long-term follow-up management

All 93 patients underwent TP were followed up through the endocrinology clinic and telephone visits. Regular follow-up by the surgeon and the oncologist was performed simultaneously. Overall survival of the TP patients with different pathologies from surgery to the date of death or last follow-up was evaluated. Diabetes assessments were available in 80 patients after TP with relatively stable general conditions and primary diseases. Follow-up variables, including HbA_1c_, fasting C-peptide, creatinine, urinary albumin-to-creatinine ratio, and liver function, were measured in patients underwent TP. Hypoglycemia data were derived from self-monitoring of blood glucose profiles. Seven TP patients underwent CGM for seven days with calibration by 4-point self-monitoring of blood glucose to obtain mean glucose value, SD, CV, time in range (TIR), time above range (TAR), and time below range (TBR), etc. Weight, gastrointestinal symptoms, pancreatic enzymes dose, and events of diabetic chronic complications were recorded.

### Comparative analysis

Additional 80 consecutive patients with complete insulin-deficient T1DM (C-peptide <0.05 ng/ml) from the endocrinology clinic between July 2020 and July 2021 were included to evaluate the differences in glycemic control and insulin therapy between patients undergoing TP and patients with T1DM. Patients with T1DM who were under the age of 18, had a BMI over 24 kg/m^2^, had eating disorders, had renal or liver dysfunction, were pregnant, were treated with steroids, or took oral antidiabetic agents were excluded.

### Statistical analysis

Data analyses were performed with SPSS version 25.0. Normally distributed continuous variables were presented as mean ± SD and analyzed with independent samples *t* test or one-way ANOVA. Nonnormally distributed continuous variables were displayed as medians (interquartile ranges) and compared using Mann-Whitney *U* test or Kruskal-Wallis *H* test. Chi-square test or Fisher’s exact test was applied for categorical variables presented as numbers (percentages). Overall survival was assessed by Kaplan-Meier analysis and compared using the logrank test. Statistical significance was considered as a two-tailed *P* value < 0.05.

## Results

### Clinical characteristics of patients undergoing TP

A total of 93 patients underwent TP were recruited with a mean age of 59.9 ± 10.8 years, of which 43 (46.2%) were male. Pancreatic malignant and benign tumors were present in 72 (77.4%) and 21 (22.6%) cases, respectively. The most common indications for TP were pancreatic ductal adenocarcinoma and IPMN (including invasive and non-invasive IPMN), accounting for 37.6% and 38.7% of all patients, respectively, followed by 8.6% of PNET and other rare pancreatic neoplasms, including serous cystadenoma, mucous cystadenocarcinoma, carcinosarcoma, ampullary carcinoma, mixed ductal-endocrine carcinoma, cholangiocarcinoma, renal cell carcinoma metastasis, and malignant fibroma metastasis. Postoperative complications occurred in 30 patients (32.3%), mainly infections (21 patients). Patients were divided into three groups according to preoperative glycemic status, including the nondiabetic group (NDG, n = 41), the short-duration diabetic group (SDG, preoperative diabetes duration ≤12 months, n = 22), and the long-duration diabetic group (LDG, preoperative diabetes duration >12 months, n = 30). Age at operation in patients of LDG was higher than those of NDG (62.9 ± 8.1 vs 57.8 ± 11.2 years, *P* = 0.038) but no significant differences in gender, pathology, postoperative complications, or postoperative length of stay among the three groups. The clinical and pathologic characteristics of TP patients are outlined in **Table 1**.

**Table 1:** Clinical and pathologic characteristics of 93 patients underwent total pancreatectomy.

Characteristics	Total	NDG	With pre-op DM		P value
			SDG	LDG	
N	93 (100)	41 (44.1)	22 (23.7)	30 (32.3)	–
Age at operation (years)	59.9 ± 10.8	57.8 ± 11.2	59.5 ± 12.6	62.9 ± 8.1^#^	0.145
Male	43 (46.2)	20 (48.8)	9 (40.9)	14 (46.7)	0.835
**Pathology**					0.829
Pancreatic carcinoma					
Ductal adenocarcinoma	35 (37.6)	14 (34.1)	11 (50.0)	10 (33.3)	–
Acinar cell carcinoma	2 (2.2)	2 (4.9)	0	0	–
Adenosquamous carcinoma	1 (1.1)	0	0	1 (3.3)	–
IPMN					
Invasive IPMN	21 (22.6)	9 (22.0)	5 (22.7)	7 (23.3)	–
Non-invasive IPMN	15 (16.1)	5 (12.2)	4 (18.2)	6 (20.0)	–
PNET*	8 (8.6)	4 (9.8)	1 (4.5)	3 (10.0)	–
Cystic neoplasms					
Serous cystadenoma	2 (2.2)	0	1 (4.5)	1 (3.3)	–
Mucous cystadenocarcinoma	1 (1.1)	1 (2.4)	0	0	–
Carcinosarcoma	2 (2.2)	2 (4.9)	0	0	–
Ampullary carcinoma	2 (2.2)	1 (2.4)	0	1 (3.3)	–
Mixed ductal-endocrine carcinoma	1 (1.1)	1 (2.4)	0	0	–
Cholangiocarcinoma	1 (1.1)	0	0	1 (3.3)	–
Renal cell carcinoma metastasis	1 (1.1)	1 (2.4)	0	0	–
Malignant fibroma metastasis	1 (1.1)	1 (2.4)	0	0	–
**Postoperative complications**	30 (32.3)	13 (31.7)	7 (31.8)	10 (33.3)	0.988
Abdominal infection	13 (14.0)	6 (14.6)	3 (13.6)	4 (13.3)	–
Hospital acquired pneumonia	6 (6.5)	3 (7.3)	3 (13.6)	0	–
Urinary infection	2 (2.2)	0	0	2 (6.7)	–
Catheter related infection	1 (1.1)	1 (2.4)	0	0	–
Abdominal hemorrhage	9 (9.7)	5 (12.2)	1 (4.5)	3 (10.0)	–
Delayed gastric emptying	2 (2.2)	1 (2.4)	1 (4.5)	0	–
Deep venous thrombosis	2 (2.2)	2 (4.9)	0	0	–
Acute coronary syndrome	2 (2.2)	0	0	2 (6.7)	–
Chyle leak	1 (1.1)	1 (2.4)	0	0	–
Postoperative length of stay (days)	14 (12, 23)	15 (13, 28)	13 (11, 19)	15 (12, 19)	0.318

Data are presented as mean ± SD or median (interquartile range) or number (percentage). pre-op, preoperative; DM, diabetes mellitus; NDG, nondiabetic group; SDG, short-duration diabetic group; LDG, long-duration diabetic group; IPMN, intraductal papillary mucinous neoplasm; PNET, pancreatic neuroendocrine tumor.

^*^Four patients with multiple endocrine neoplasia type 1.

^#^P < 0.05 vs NDG.

### Perioperative period

#### Preoperative glycemic status

Of the 93 patients, diabetes was present in 52 (55.9%) patients before surgery, and 20 (38.5%) of the 52 had already received insulin therapy. Patients of LDG had higher BMI and proportion of diabetic family history than those of SDG (23.13 ± 3.57 vs 21.27 ± 2.82 kg/m^2^ and 46.7% vs 13.6%, respectively; *P* < 0.05). As more patients in LDG had received antidiabetic therapy (96.7% vs 63.6%, *P* = 0.003), individuals in LDG exhibited lower HbA_1c_ and 2-hour postprandial blood glucose than those in SDG (6.95 ± 1.15 vs 8.09 ± 1.95% and 11.55 ± 3.63 vs 15.87 ± 5.78 mmol/L, respectively; *P* < 0.01). None was complicated with diabetic retinopathy or diabetic kidney disease, while five patients with a median preoperative diabetes duration of 5 years had coronary heart disease. The features categorized by preoperative glycemic status are summarized in **Table 2**.

**Table 2:** Perioperative glucose measurements and insulin regimens in patients underwent total pancreatectomy.

Characteristics	Total	NDG	With pre-op DM		P value	
			SDG	LDG		
N, n (%)	93 (100)	41 (44.1)	22 (23.7)	30 (32.3)	–
**Preoperative glycemic status**					
Age at operation (years), mean ± SD	59.9 ± 10.8	57.8 ± 11.2^c^	59.5 ± 12.6	62.9 ± 8.1^a^	0.145
BMI (kg/m^2^), mean ± SD	22.06 ± 3.13	21.70 ± 2.79	21.27 ± 2.82^c^	23.13 ± 3.57^b^	0.063
DM duration (months), median (IQR)	36 (6, 60)	NA	5 (1, 12)^c^	60 (36, 120)^b^	**< 0.001**
Family history of DM, n (%)	18 (19.4)	1 (2.4)^c^	3 (13.6)^c^	14 (46.7)^ab^	**< 0.001**
FBG (mmol/L), mean ± SD	7.02 ± 2.16	5.72 ± 0.81^bc^	7.91 ± 2.54^a^	7.93 ± 2.21^a^	**< 0.001**
2-h PBG (mmol/L), mean ± SD	12.67 ± 5.07	5.30 ± 0.42^bc^	15.87 ± 5.78^ac^	11.55 ± 3.63^ab^	**< 0.001**
HbA_1c_ (%), mean ± SD	7.24 ± 1.72	5.48 ± 0.77^bc^	8.09 ± 1.95^ac^	6.95 ± 1.15^ab^	**0.007**
Antidiabetic medications, n (%)	43 (46.2)	–	14 (63.6)^c^	29 (96.7)^b^	**0.003**
Oral agents only, n (%)	23 (24.7)	–	7 (31.8)	16 (53.3)	0.162
Insulin-containing regimes, n (%)	20 (21.5)		7 (31.8)	13 (43.3)	0.565
Daily insulin dose (units/kg/day), mean ± SD	0.53 ± 0.28	–	0.42 ± 0.24	0.59 ± 0.29	0.224
CAD, n (%)	7 (7.5)	2 (4.9)	0	5 (16.7)	0.056
Stroke, n (%)	4 (4.3)	0	1 (4.5)	3 (10.0)	0.080
DKD, n (%)	–	–	0	0	–
DR, n (%)	–	–	0	0	–
**Postoperative hospitalized glucose measurement**					
Mean BG (mmol/L), mean ± SD	10.99 ± 1.13	10.96 ± 1.06	11.23 ± 1.09	10.86 ± 1.25	0.497
Maximum BG (mmol/L), mean ± SD	21.75 ± 3.19	21.80 ± 3.08	21.68 ± 2.90	21.72 ± 3.63	0.989
Minimum BG (mmol/L), mean ± SD	4.25 ± 1.11	4.02 ± 1.06	4.68 ± 1.29	4.22 ± 0.96	0.077
6 am BG (mmol/L), mean ± SD	10.87 ± 1.97	10.62 ± 1.73	11.50 ± 2.43	10.74 ± 1.88	0.218
CV (%), mean ± SD	31.54 ± 5.42	32.50 ± 4.94	30.31 ± 5.10	31.13 ± 6.19	0.281
Within the target range (4.4-10 mmol/L), n (%)	2698 (43.3)	1211 (43.0)	569 (41.4)	918 (45.1)	0.101
Hyperglycemic events					
10.1-13.9 mmol/L, n (%)	1976 (31.7)	894 (31.8)	450 (32.8)	632 (31.0)	0.560
14.0-16.7 mmol/L, n (%)	775 (12.5)	358 (12.7)	180 (13.1)	237 (11.6)	0.367
> 16.7 mmol/L, n (%)	610 (9.8)	270 (9.6)	147 (10.7)	193 (9.5)	0.437
Hypoglycemic events					
3-3.8 mmol/L, n (%)	83 (1.3)	42 (1.5)	14 (1.0)	27 (1.3)	0.473
< 3 mmol/L, n (%)	10 (0.2)	7 (0.2)	0	3 (0.1)	0.156
Patients with hypoglycemic events, n of patients/total n (%)	42/93 (45.2)	21/41 (51.2)	9/22 (40.9)	12/30 (40.0)	0.620
3-3.8 mmol/L, n of patients/total n (%)	38/93 (40.9)	19/41 (46.3)	9/22 (40.9)	10/30 (33.3)	0.547
< 3 mmol/L, n of patients/total n (%)	8/93 (8.6)	5/41 (12.2)	0	3/30 (10.0)	0.311
**Postoperative insulin regimen**					
Parenteral nutrition					
Daily insulin dose (units/kg/day), mean ± SD	1.20 ± 0.47	1.07 ± 0.40^c^	1.13 ± 0.34^c^	1.45 ± 0.56^ab^	**0.012**
Daily insulin dose (units/day), mean ± SD	69.03 ± 32.53	60.33 ± 27.55^c^	63.32 ± 23.74^c^	85.09 ± 38.79^ab^	**0.003**
Insulin dose per 10g carbohydrate (units), mean ± SD	5.70 ± 2.56	5.11 ± 2.02^c^	4.93 ± 1.80^c^	7.10 ± 3.15^ab^	**0.008**
Enteral nutrition					
Daily insulin dose (units/kg/day), mean ± SD	0.36 ± 0.13	0.35 ± 0.16	0.34 ± 0.11	0.40 ± 0.11	0.267
Daily insulin dose (units/day), mean ± SD	21.19 ± 8.98	20.30 ± 9.90	18.77 ± 7.22	24.05 ± 8.37	0.107
1-month post-op					
Daily insulin dose (units/kg/day), mean ± SD	0.38 ± 0.12	0.36 ± 0.11^c^	0.35 ± 0.12^c^	0.42 ± 0.12^ab^	**0.031**
Daily insulin dose (units/day), mean ± SD	21.42 ± 8.01	20.22 ± 7.96^c^	18.89 ± 6.98^c^	24.91 ± 7.82^ab^	**0.011**
Basal percentage (%), median (IQR)	40.0 (32.1, 48.7)	40.0 (29.4, 47.7)	40.0 (33.3, 62.9)	36.1 (30.8, 44.7)	0.573

pre-op, preoperative; DM, diabetes mellitus; NDG, nondiabetic group; SDG, short-duration diabetic group; LDG, long-duration diabetic group; BMI, body mass index; FBG, fasting blood glucose; PBG, postprandial blood glucose; HbA_1c_, glycosylated hemoglobin A_1c_; CAD, coronary artery disease; DKD, diabetic kidney disease; DR, diabetic retinopathy; BG, blood glucose; CV, coefficient of variation; post-op, postoperation.

^a^P < 0.05 vs NDG. ^b^P < 0.05 vs SDG. ^c^P < 0.05 vs LDG.

P values < 0.05 were shown in bold.

#### Postoperative hospitalized glucose measurement

A total of 6224 blood glucose measurements were performed in a median of 14 days postoperative hospital stay. The mean blood glucose was 10.99 ± 1.13 mmol/L, and the 6 am blood glucose was 10.87 ± 1.97 mmol/L. Glucose within the target range (4.4-10.0 mmol/L) and in the range of 4.4-13.9 mmol/L accounted for 43.3% and 75.1% of all glucose data, respectively. The CV calculated from bedside blood glucose monitoring was 31.54 ± 5.42%. Hypoglycemic events occurred in 42 patients (45.2%), accounting for 1.5% of all glucose data, mainly of 3.0 to 3.8mmol/L. There was no significant difference among the three groups of NDG, SDG, and LDG with regard to mean blood glucose, maximum blood glucose, minimum blood glucose, 6 am blood glucose, CV, glucose within the target range, and the proportion of hyperglycemic and hypoglycemic events (all *P* > 0.05). The detailed postoperative hospitalized glucose measurements are listed in **Table 2**.

#### Postoperative insulin regimen

**Figure 1:**
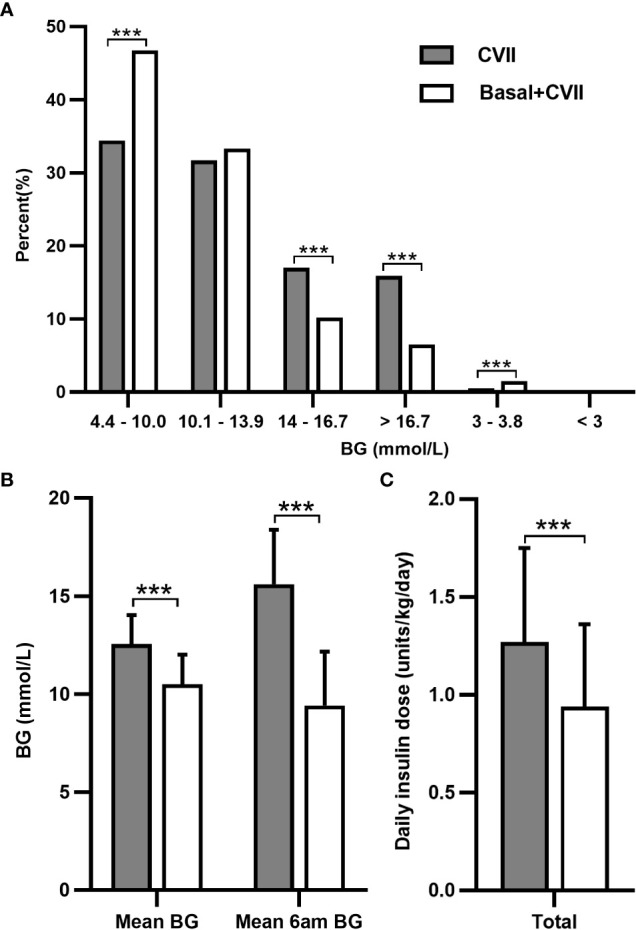
Analysis of the glycemic control and daily insulin dose after adding basal insulin to CVII therapy in 46 patients after total pancreatectomy during postoperative parenteral nutrition. Changes in the percentage of glucose data **(A)**, mean BG, mean 6 am BG **(B)**, and daily insulin dose **(C)**. CVII, continuous intravenous insulin infusion. BG, blood glucose. Data are presented as mean ± SD. ***P < 0.001.

During the first few days after surgery, TPN with a median carbohydrate content of 120 (100, 142) g was given for 12 to 15 hours per day. The daily insulin dose was 1.20 ± 0.47 units/kg/day (69.03 ± 32.53 units/day), and the insulin dose per 10g carbohydrate was 5.70 ± 2.56 units, which were all higher in individuals of LDG than in those of NDG (1.45 ± 0.56 vs 1.07 ± 0.40 units/kg/day, 85.09 ± 38.79 vs 60.33 ± 27.55 units/day, and 7.10 ± 3.15 vs 5.11 ± 2.02 units, respectively; *P* < 0.05) and SDG (1.45 ± 0.56 vs 1.13 ± 0.34 units/kg/day, 85.09 ± 38.79 vs 63.32 ± 23.74 units/day, and 7.10 ± 3.15 vs 4.93 ± 1.80 units, respectively; *P* < 0.05). No significant changes were found in insulin dose between NDG and SDG (**Table 2**). Subsequently, a mean long-acting insulin Lantus dose of 0.16 ± 0.58 units/kg/day was added on the basis of CVII therapy in 46 patients with a longer duration of parenteral nutrition. After the combination of basal insulin, the percentage of glucose within the target range (4.4-10.0 mmol/L) increased (34.4% vs 46.7%, *P* < 0.001), and that of hyperglycemic events decreased with a slight increase in level 1 hypoglycemia (3.0-3.8 mmol/L) (0.5% vs 1.5%, *P* < 0.001) but no change in level 2 hypoglycemia (< 3.0mmol/L) (**Figure 1A**). Mean blood glucose (12.55 ± 1.48 vs 10.50 ± 1.52 mmol/L, *P* < 0.001) significantly decreased, as well as mean 6 am blood glucose (15.61 ± 2.78 vs 9.42 ± 2.74 mmol/L, *P* < 0.001) (**Figure 1B**) and daily insulin dose (1.27 ± 0.48 vs 0.94 ± 0.42 units/kg/day, *P* < 0.001) (**Figure 1C**).

**Figure 2:**
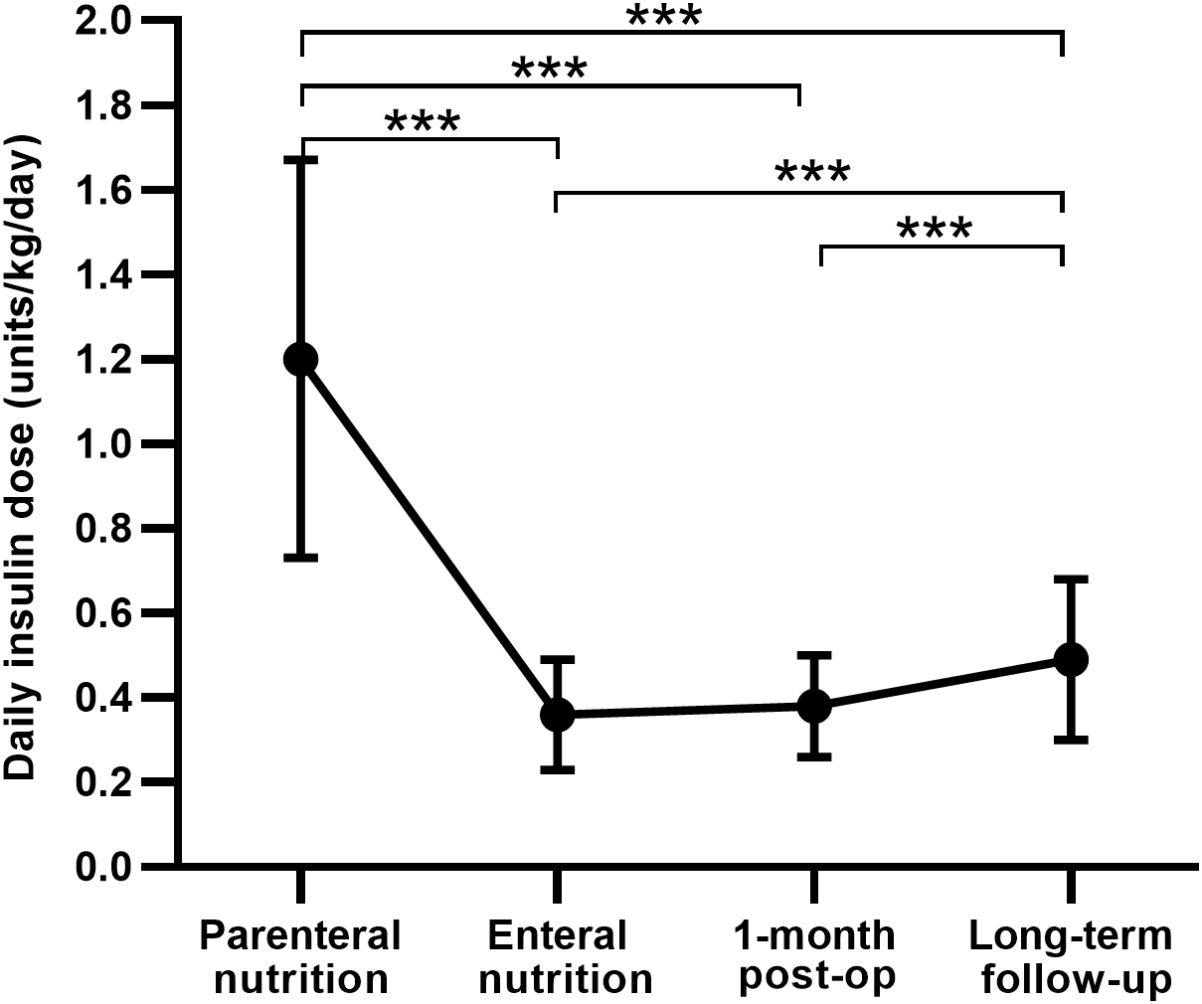
Comparison of the daily insulin dose between different periods after total pancreatectomy. post-op, postoperation. Data are presented as mean ± SD. ***P < 0.001.

The Insulin regimen was switched from CVII to multiple daily insulin injections (MDI) along with the recovery of enteral nutrition, and the daily insulin dose was significantly reduced to 0.36 ± 0.13 units/kg/day. At one month after TP, the daily insulin dose was 0.38 ± 0.12 units/kg/day, similar to the insulin dose in the enteral nutrition period, and was also higher in patients of LDG than in those of NDG (0.42 ± 0.12 vs 0.36 ± 0.11 units/kg/day, *P* = 0.021) and SDG (0.42 ± 0.12 vs 0.35 ± 0.12 units/kg/day, *P* = 0.027). See **Table 2** and **Figure 2** for details.

### Long-term follow-up period

#### Overall survival of TP

**Figure 3:**
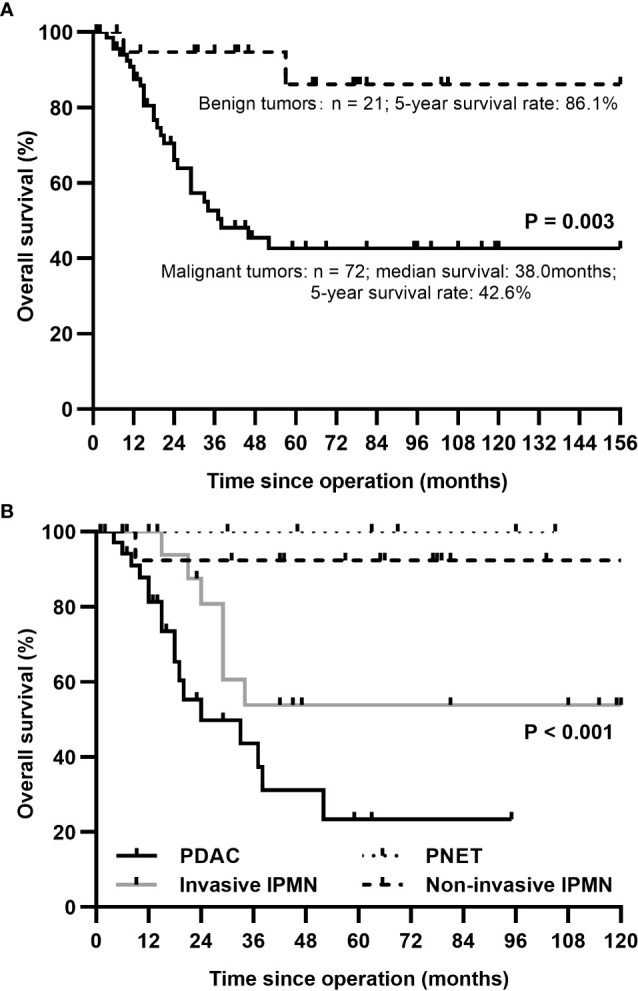
The Kaplan-Meier survival curve for patients after total pancreatectomy according to underlying diseases. **(A)** A better survival rate was identified in benign tumors compared with malignant tumors. **(B)** 1-year survival rates for patients with PDAC, invasive IPMN, non-invasive IPMN, and PNET: 81.2%, 100%, 92.3%, 100%; 3-year: 43.5%, 53.8%, 92.3%, 100%; 5-year: 23.3%, 53.8%, 92.3%, 100%. PDAC, pancreatic ductal adenocarcinoma; IPMN, intraductal papillary mucinous neoplasm; PNET, pancreatic neuroendocrine tumor.

The 5-year survival rates for patients with malignant and benign tumors were 42.6% and 86.1%, respectively. In the subgroup analysis, patients with ductal adenocarcinoma had a median survival of 24.0 months and the 1-, 3-, and 5-year survival rates of 81.2%, 43.5%, and 23.3%, respectively. Pancreatic ductal adenocarcinoma had the lowest 5-year survival rate (23.3%), followed by invasive IPMN (53.8%), while non-invasive IPMN and PNET had favorable 5-year survival rates of 92.3% and 100%, respectively. The estimated long-term overall survival following TP according to pathology is displayed in **Figure 3**.

#### Glycemic level and variability of patients undergoing TP

At a median follow-up of 20 months after TP, patients all recovered normal food intake, and 90.3% were free of fatty diarrhea with a median pancreatic enzyme dosage of 900 (900, 1350) mg per day. However, a mean weight loss of 4.50 (95% confidence interval, 3.21-5.80) kg was still observed in 69.9% of patients compared to preoperative weight. Serum C-peptide was undetectable, and HbA_1c_ was 7.43 ± 0.76%. During the one month before the last follow-up, 47 patients (58.8%) experienced at least one hypoglycemic episode. The respective values for mean glucose levels, SD, TIR, and CV assessed by CGM were 8.61 ± 1.59 mmol/L, 3.27 ± 0.71 mmol/L, 71.92 ± 9.43%, and 37.49 ± 8.26%. Other CGM measurements are presented in **Table 3**.

**Table 3:** Comparison of characteristics of diabetes mellitus after total pancreatectomy and type 1 diabetes in the long-term follow-up period.

Characteristics	TP(n = 80)	T1DM(n = 80)	P value
Age at last follow-up (years), mean ± SD	62.59 ± 10.41	44.01 ± 17.22	**< 0.001**
Male, n (%)	36 (45)	30 (37.5)	0.422
Diabetes duration (months), median (IQR)	20 (7, 63)*	132 (72, 204)	**< 0.001**
BMI (kg/m^2^), mean ± SD	20.33 ± 2.39	20.92 ± 1.76	0.079
HbA_1c_ (%), mean ± SD	7.43 ± 0.76	7.66 ± 1.12	0.128
C-peptide (ng/ml), median (IQR)	<0.05	<0.05	–
Hypoglycemic events, n of patients/total n (%)	47/80 (58.8)	57/80 (71.3)	0.097
**CGM measurements**†			
Mean glucose value (mmol/L), mean ± SD	8.61 ± 1.59	8.25 ± 1.62	0.665
SD (mmol/L), mean ± SD	3.27 ± 0.71	3.24 ± 1.22	0.967
CV (%), mean ± SD	37.49 ± 8.26	38.22 ± 6.94	0.851
Maximum glucose value (mmol/L), mean ± SD	18.80 ± 3.51	17.71 ± 2.56	0.491
Minimum glucose value (mmol/L), mean ± SD	2.93 ± 0.56	3.20 ± 0.69	0.396
TIR (%) (3.9-10 mmol/L), mean ± SD	71.92 ± 9.43	70.19 ± 14.99	0.787
TAR (%) (>10 mmol/L), mean ± SD	23.41 ± 9.78	24.38 ± 14.51	0.877
TBR (%) (<3.9 mmol/L), mean ± SD	4.68 ± 4.54	5.42 ± 3.13	0.707
**Insulin regimen**			
Daily insulin dose (units/kg/day), mean ± SD	0.49 ± 0.19	0.65 ± 0.19	**< 0.001**
Daily insulin dose (units/day), mean ± SD	27.09 ± 11.88	38.74 ± 11.44	**< 0.001**
Basal percentage (%), mean ± SD	39.4 ± 16.5	43.9 ± 9.9	**0.035**
**Insulin pump**‡			
Daily insulin dose (units/kg/day), mean ± SD	0.44 ± 0.14	0.66 ± 0.14	**< 0.001**
Daily insulin dose (units), mean ± SD	24.81 ± 8.05	39.93 ± 10.22	**< 0.001**
Basal percentage (%), mean ± SD	44.1 ± 14.5	49.8 ± 8.86	0.138

TP, total pancreatectomy; T1DM, type 1 diabetes mellitus; BMI, body mass index; HbA_1c_, glycosylated hemoglobin A_1c_; CGM, continuous glucose monitoring; SD, standard deviation of glucose; CV, coefficient of variation; TIR, time in range; TAR, time above range; TBR, time below range.

^*^Diabetes duration since total pancreatectomy.

†The CGM data were available in seven patients undergoing TP and eight with T1DM.

‡Insulin pumps were used in 14 patients after TP and 24 patients with T1DM.

P values < 0.05 were shown in bold.

Compared with patients with complete insulin-deficient T1DM, TP patients were older (62.59 ± 10.41 vs 44.01 ± 17.22 years, *P* < 0.001) with shorter diabetes duration (20 vs 132 months, *P* < 0.001) but similar BMI (20.33 ± 2.39 vs 20.92 ± 1.76 kg/m^2^, *P* = 0.079). Comparative analysis revealed no significant difference in HbA_1c_ (7.43 ± 0.76 vs 7.66 ± 1.12%, *P* = 0.128) and proportion of patients experiencing hypoglycemic events (58.8 vs 71.3%, *P* = 0.097) between these two groups. The mean glucose value, TIR, TAR, and TBR obtained by CGM were comparable between TP patients and patients with T1DM (8.61 ± 1.59 vs 8.25 ± 1.62 mmol/L, 71.92 ± 9.43 vs 70.19 ± 14.99%, 23.41 ± 9.78 vs 24.38 ± 14.51%, and 4.68 ± 4.54 vs 5.42 ± 3.13%, respectively; *P* > 0.05). The glycemic variability indices, including SD and CV, did not differ significantly among the two groups either (3.27 ± 0.71 vs 3.24 ± 1.22 mmol/L and 37.49 ± 8.26 vs 38.22 ± 6.94%, respectively; *P* > 0.05). **Table 3** provides a summary of the comparison outcomes.

#### Diabetes treatment for patients undergoing TP

All 80 patients undergoing TP were treated with insulin, 63 of them (78.8%) received the MDI regimen, and only three patients (3.8%) were on premixed insulin injections. Daily insulin dose increased significantly at a median follow-up length of 20 months in comparison with one month after operation (0.49 ± 0.19 vs 0.38 ± 0.12 units/kg/day, *P* < 0.001) (**Figure 2**), with a mean basal insulin proportion of 39.4%. Comparison of insulin treatment between TP and T1DM showed that the daily insulin dose and the basal insulin percentage of patients with T1DM was higher than patients after TP (0.65 ± 0.19 vs 0.49 ± 0.19 units/kg/day, 38.74 ± 11.44 vs 27.09 ± 11.88 units/day and 43.9 ± 9.9 vs 39.4 ± 16.5%, respectively; *P* < 0.05) (**Table 3**). In subgroup analysis, with comparable postoperative BMI and HbA_1c_ among the three groups, insulin requirements were higher in LDG than in NDG (0.60 ± 0.21 vs 0.42 ± 0.12 units/kg/day, *P* < 0.001) and SDG (0.60 ± 0.21 vs 0.42 ± 0.17 units/kg/day, *P* = 0.003), but were equivalent in the latter two groups. While the daily insulin dose was lower in NDG and SDG than in T1DM (0.42 ± 0.12 vs 0.65 ± 0.19 and 0.42 ± 0.17 vs 0.65 ± 0.19 units/kg/day, respectively; *P* < 0.001), it was similar between LDG and T1DM (0.60 ± 0.21 vs 0.65 ± 0.19 units/kg/day, *P* = 0.295). Glycemic control and treatment in the long-term follow-up period classified by preoperative glycemic status are shown in **Table 4**.

**Table 4:** Glycemic control and treatment of patients undergoing total pancreatectomy in long-term follow-up period categorized by preoperative glycemic status.

Characteristics	Total	NDG	With pre-op DM		P value
			SDG	LDG	
N	80 (100)	34 (42.5)	17 (21.3)	29 (36.3)	–
Age at last follow-up (years)	62.59 ± 10.41	60.53 ± 11.19^c^	61.29 ± 12.10	65.76 ± 7.61^a^	0.079
BMI (kg/m^2^)	20.33 ± 2.39	20.00 ± 2.31	20.03 ± 2.45	20.89 ± 2.44	0.290
HbA_1c_ (%)	7.43 ± 0.76	7.32 ± 0.83	7.77 ± 0.88	7.36 ± 0.52	0.193
**Insulin regimen**					0.411
MDI	63 (78.8)	28 (82.4)	12 (70.6)	23 (79.3)	–
Premixed insulin	3 (3.8)	0	2 (11.8)	1 (3.4)	–
Insulin pump	14 (17.5)	6 (17.6)	3 (17.6)	5 (17.2)	–
Daily insulin dose (units/kg/day)	0.49 ± 0.19	0.42 ± 0.12^c^	0.42 ± 0.17^c^	0.60 ± 0.21^ab^	**< 0.001**
Daily insulin dose (units/day)	27.09 ± 11.88	23.00 ± 7.57^c^	22.98 ± 9.41^c^	34.28 ± 14.00^ab^	**< 0.001**
Basal percentage (%)	39.4 ± 16.4	37.2 ± 16.6	45.6 ± 17.4	38.4 ± 15.3	0.208
**Insulin pump**					
N	14 (100)	9 (64.3)*		5 (35.7)	–
Daily insulin dose (units/kg/day)	0.44 ± 0.14	0.38 ± 0.10		0.53 ± 0.15	**0.043**
Daily insulin dose (units/day)	24.81 ± 8.05	22.02 ± 6.97		29.83 ± 8.02	0.081
Basal percentage (%)	44.1 ± 14.5	43.4 ± 13.5		45.9 ± 17.2	0.743
**Combined oral antidiabetic agent**	7 (8.8)	1 (2.9)	1 (5.9)	5 (17.2)	0.222
Metformin	3 (3.8)	0	0	3 (10.3)	–
α-glucosidase inhibitors	4 (5.0)	1 (2.9)	1 (5.9)	2 (6.9)	–

Data are presented as mean ± SD or number (percentage).

pre-op, preoperative; DM, diabetes mellitus; NDG, nondiabetic group; SDG, short-duration diabetic group; LDG, long-duration diabetic group; BMI, body mass index; HbA_1c_, glycosylated hemoglobin A_1c_; MDI, multiple daily insulin injections.

*Including seven preoperative nondiabetic patients and two short-duration diabetic patients.

^a^P < 0.05 vs NDG. ^b^P < 0.05 vs SDG. ^c^P < 0.05 vs LDG.

P values < 0.05 were shown in bold.

**Figure 4:**
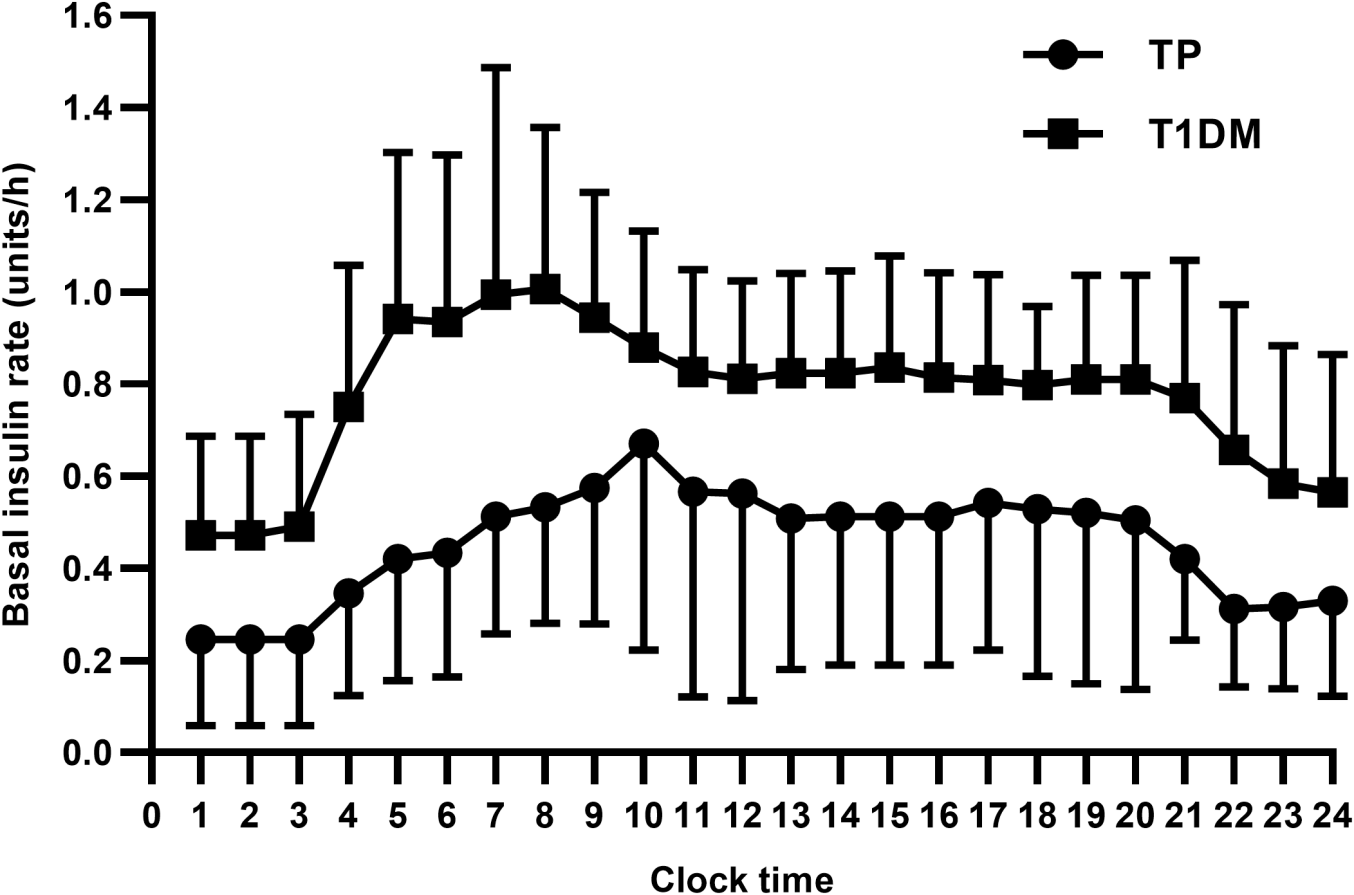
TP, total pancreatectomy; T1DM, type 1 diabetes mellitus. Data are presented as mean ± SD. All *P* values were less than 0.05.

Fourteen patients used insulin pump therapy with a daily insulin dose of 0.44 ± 0.14 units/kg/day and a mean basal insulin proportion of 44.1%. Among them, five patients of LDG required more insulin than patients of NDG or SDG (0.53 ± 0.15 vs 0.38 ± 0.10 units/kg/day, *P* = 0.043) (**Table 4**). In patients on insulin pump therapy, daily insulin dose was also lower in patients undergoing TP than in those with T1DM (0.44 ± 0.14 vs 0.66 ± 0.14 units/kg/day and 24.81 ± 8.05 vs 39.93 ± 10.22 units, *P* < 0.001) (**Table 3**). The basal insulin infusion rates at all time points of TP patients were significantly lower than patients with T1DM (all *P* < 0.05). An increasing trend of insulin infusion rate in the morning could be observed in both TP and T1DM patients (**Figure 4**). However, no discernible difference was found between the MDI and continuous subcutaneous insulin infusion (CSII) group in terms of HbA_1c_, the proportion of hypoglycemia events, and insulin dose (7.42 ± 0.74 vs 7.49 ± 0.84%, 59.1 vs 57.1% and 0.49 ± 0.20 vs 0.44 ± 14 units/kg/day, *P* > 0.05).

Seven patients received oral antidiabetic agents in combination with insulin therapy, including three patients treated with metformin and four with α-glycosidase inhibitors. Five of the seven patients were from LDG (**Table 4**).

#### Diabetic complications of patients undergoing TP

Two out of eighty patients developed microalbuminuria (urinary albumin-to-creatinine ratio 30-300 mg/g) with normal creatinine levels at 119 months and 96 months after TP, respectively. There was no diabetic retinopathy in any of the 16 patients who underwent ophthalmofundoscopy. All the patients were free of nonfatal myocardial infarction and stroke events throughout the follow-up. No patient needed medical intervention owing to diabetic ketoacidosis, and no deaths in this series were attributed to diabetic complications.

## Discussion

This large cohort presented a comprehensive picture of glycemic control, insulin therapy, diabetic complications, and surgical outcomes in patients undergoing TP from perioperative to long-term follow-up periods, providing reference protocols for CVII, MDI, and CSII at different postoperative stages.

During hospitalization after TP, the proportion of glucose value within the target range (4.0-10.0 mmol/L) was consistent with the previous research (17), and the hypoglycemic events were rare under close glucose monitoring. Compared with the common insulin dose during the TPN period for patients with diabetes (16), the higher ratio at 5.70 units of regular insulin per 10 g carbohydrate following TP might be attributed to complete insulin deficiency, postoperative stress, and the lack of basal insulin. After adding basal insulin to CVII therapy, the daily insulin dose decreased, and glycemic control further improved with an acceptable hypoglycemia risk, indicating that timely and adequate basal insulin replacement was important during the postoperative parenteral period after TP. From our experience, subcutaneous basal insulin can be administered at 0.1-0.2 units/kg/day on the basis of CVII and adjusted until fasting blood glucose was appropriate. Pre-meal rapid-acting insulin can be given according to the food intake and blood glucose values.

Andersen et al. (17) reported that parenteral nutrition with insulin treatment after TP improved glycemic control compared with glucose infusion and reduced non-infectious postoperative complications. In our cohort, all patients were given TPN after surgery, and the insulin dose decreased after the gradual transition to enteral nutrition. Guidelines for nutrition support therapy suggest that once tolerance to enteral nutrition improves, the amount of parenteral nutrition should be reduced and discontinued when the patient receives >60% of total energy from enteral nutrition to minimize the negative effect of hyperglycemia (18). Further research can be done about the impact of parenteral and enteral nutrition on blood glucose and surgical complications in patients undergoing TP.

During the long-term follow-up, we noted the mean HbA_1c_ level of 7.43% after TP was in accordance with recent larger cohorts, which ranged from 7.3% to 7.9% (10, 19–23). Several previous studies reported the incidence of hypoglycemia after TP ranging from 42.0% to 100.0% with a median of 2 occurrences per week or 10 per month (10, 24–28), and body weight loss, low total cholesterol level, strict glycemic control, and using rapid-acting insulin were risk factors for hypoglycemia (28). We performed a comprehensive evaluation of glycemic control through CGM, confirming great glycemic variability in patients after TP but comparable mean glucose values, TIR, TBR, TAR, and CV to patients with complete insulin-deficient T1DM. Juel et al. (29) also reported similar CV and TBR assessed by CGM between TP and T1DM patients but lower TIR, higher TAR, and higher continuous overall net glycemic action per 60 minutes were observed in TP patients. Furthermore, HbA_1c_, hospitalization rate secondary to hypoglycemia, and impact of diabetes after TP on most domains in quality of life were paralleled to insulin-dependent diabetes from other causes (13, 30–33). Overall, glycemic control after TP can be similar to T1DM under regular follow-up but indeed is influenced by more factors, such as diet recovery, pancreatic enzyme supplementation, and primary disease treatment.

With the recovery of food intake and sufficient pancreatin replacement, the mean daily insulin dose at long-term follow-up considerably increased compared to one month after surgery. The mean daily insulin dose was 27.09 ± 11.88 units/day (0.49 ± 0.19 units/kg/day), which was in parallel with that in other published studies varying from 23 to 37 units (8, 10, 23–25, 34, 35) or 0.50 to 0.58 units/kg/day (15, 21, 23, 34). Our findings, which were also in line with the previous research, showed a marked decrease in daily insulin requirements and basal insulin percentages in patients after TP compared to T1DM with similar BMI and complete insulin deficiency (36, 37). Moreover, in subgroup analysis compared with T1DM, the decrease in insulin dose was observed only in patients of NDG and SDG but not LDG. The lower daily and basal insulin needs in patients following TP may be attributed to increased peripheral insulin sensitivity, malabsorption, and defect in the counterregulatory mechanism offered by pancreatic glucagon (36, 38).

Patients with preoperative long-duration diabetes had higher daily insulin requirements than those without diabetes or those with preoperative short-duration diabetes during both perioperative and long-term follow-up periods. This phenomenon may reflect that patients with preoperative long-duration diabetes have a certain degree of insulin resistance. The patients with preoperative diabetes all had extensive involvement of the pancreas, making it difficult to distinguish between type 2 diabetes and pancreatogenic diabetes preoperatively. Higher age, BMI, and proportion of diabetes family history were also characteristics of patients with preoperative long-duration diabetes, which could help determine whether patients have greater insulin requirements. Therefore, preoperative identification of glycemic status and diabetes classification in patients planned to undergo TP are important to guide postoperative insulin treatment.

In our cohort, TP patients who received insulin pump therapy had similar daily insulin requirements but higher basal insulin percentages compared to the previous literature (36). The increase in insulin infusion rate early in the morning was also detected in TP patients, suggesting that the dawn phenomenon also existed in patients after TP despite the deficiency of pancreatic glucagon. The dawn phenomenon after TP may derive from other counter-regulatory hormones rather than the pancreatic glucagon, as the levels of cortisol, thyroxine, and growth hormone have been reported to be comparable between patients after TP and patients with T1DM (36). We identified no difference in glycemic control or insulin dose between the CSII group and the MDI group, but Struyvenberg et al. (26) demonstrated a significant reduction in severe hypoglycemic events in the CSII group than in the MDI group. Additionally, it was reported that artificial pancreas, sensor-augmented predictive low-glucose suspend pump and advanced hybrid closed−loop systems were efficacious and safe for perioperative and long-term glycemic control after TP (39–41). Hence, advanced techniques in diabetes care, including CGM, CSII, and new insulin preparations, can potentially allow better glycemic control in patients after TP. However, prospective, randomized controlled clinical trials were still needed.

Only a few patients in our cohort received combined oral antidiabetic agents after TP. Several studies have reported other antidiabetic medications for patients after TP. Juel et al. (42) disclosed that glucagon-like peptide 1 receptor agonist lixisenatide reduced postprandial plasma glucose excursions in TP patients by decelerating gastric emptying and reducing postprandial responses of gut-derived glucagon. A case report showed that the suppression of extrapancreatic glucagon by octreotide long-acting repeatable improved the hyperglycemia in a TP patient with PNET (43).

Diabetic complications after TP should be taken seriously because patients with benign diseases have a long-life expectancy, as shown in the survival analysis of our cohort. A systematic review of outcomes after TP reported no diabetes-related mortality since 2005 and rare diabetic ketoacidosis (9). The risk for microvascular complications of pancreatogenic diabetes appeared to be similar to other types of diabetes (44). In a mayo clinic cohort, end-organ complications after TP developed in 28% of patients during a mean follow-up of 3.8 years (34). Crippa et al. (35) reported diabetic complications in 6 of 45 patients at least 60 months follow-up after TP, including four patients with peripheral vascular disease, one stroke, and one retinopathy. In our study, we observed the development of microalbuminuria in only 2 out of 80 individuals without new-onset cardiovascular disease. The diabetic outcomes after TP require longer follow-ups to clarify.

The present study had some limitations. Firstly, the perioperative data were collected retrospectively, so the insulin adjustment protocol at hospitalization was not completely consistent. In the second place, several patients with malignant tumors received adjuvant chemotherapy, tyrosine kinase inhibitors, or somatostatin analog during follow-up, which might have an adverse effect on glycemic control. Thirdly, the selected patients with different pancreatic pathologies increased the heterogeneity of subjects. Finally, longer follow-ups are needed for the perioperative and long-term outcomes. Further prospective randomized controlled trials are required to determine the optimal treatment and glucose targets for patients after TP.

## Conclusions

In conclusion, glycemic control following TP could be kept within an acceptable range. Patients undergoing TP usually had high insulin requirements in the postoperative parenteral nutrition period. During long-term follow-up, similar glycemic control and variability but lower insulin needs were observed in patients after TP compared to those with complete insulin-deficient T1DM. Considering preexisting long-duration diabetes was associated with higher insulin requirements postoperatively, we proposed that preoperative glycemic status should be evaluated as it could guide insulin treatment after TP. In addition, both perioperative and long-term multidisciplinary management, including primary disease treatment, intensive diabetes care, adequate pancreatic enzyme supplementation, and nutritional support, have an essential role in improving the short- and long-term outcomes of TP.

## Data availability statement

The raw data supporting the conclusions of this article will be made available by the authors, without undue reservation.

## Ethics statement

The studies involving human participants were reviewed and approved by the Ethics Committee of Peking Union Medical College Hospital. The patients/participants provided their written informed consent to participate in this study.

## Author contributions

TYZ, WZ, and TY designed the study. TYZ, WZ, TY, YF, TPZ, JG, QL, SS, YD, and YG performed clinical evaluation and management for patients. TYZ, WZ, TY, and YF collected the clinical data. TYZ conducted the statistical analysis and drafted the manuscript. WZ and TY revised the manuscript. All authors contributed to the article and approved the submitted version.
